# Inducible *Wnt16* inactivation: WNT16 regulates cortical bone thickness in adult mice

**DOI:** 10.1530/JOE-18-0020

**Published:** 2018-03-12

**Authors:** Claes Ohlsson, Petra Henning, Karin H Nilsson, Jianyao Wu, Karin L Gustafsson, Klara Sjögren, Anna Törnqvist, Antti Koskela, Fu-Ping Zhang, Marie K Lagerquist, Matti Poutanen, Juha Tuukkanen, Ulf H Lerner, Sofia Movérare-Skrtic

**Affiliations:** 1 Centre for Bone and Arthritis ResearchDepartment of Internal Medicine and Clinical Nutrition, Institute of Medicine, Sahlgrenska Academy, Gothenburg, Sweden; 2 Department of Anatomy and Cell BiologyInstitute of Cancer Research and Translational Medicine, Faculty of Medicine, University of Oulu, Oulu, Finland; 3 Research Centre for Integrative Physiology and PharmacologyTurku Center for Disease Modeling, Institute of Biomedicine, University of Turku, Turku, Finland

**Keywords:** cortical thickness, WNT16, tamoxifen, transgenic

## Abstract

Substantial progress has been made in the therapeutic reduction of vertebral fracture risk in patients with osteoporosis, but non-vertebral fracture risk has been improved only marginally. Human genetic studies demonstrate that the *WNT16* locus is a major determinant of cortical bone thickness and non-vertebral fracture risk and mouse models with life-long *Wnt16* inactivation revealed that WNT16 is a key regulator of cortical thickness. These studies, however, could not exclude that the effect of *Wnt16* inactivation on cortical thickness might be caused by early developmental and/or growth effects. To determine the effect of WNT16 specifically on adult cortical bone homeostasis, *Wnt16* was conditionally ablated in young adult and old mice through tamoxifen-inducible Cre-mediated recombination using CAG-Cre-ER; *Wnt16*^flox/flox^ (*Cre-Wnt16*^flox/flox^) mice. First, 10-week-old *Cre-Wnt16*^flox/flox^ and *Wnt16*^flox/flox^ littermate control mice were treated with tamoxifen. Four weeks later, *Wnt16* mRNA levels in cortical bone were reduced and cortical thickness in femur was decreased in *Cre-Wnt16*^flox/flox^ mice compared to *Wnt16*^flox/flox^ mice. Then, inactivation of *Wnt16* in 47-week-old mice (evaluated four weeks later) resulted in a reduction of *Wnt16* mRNA levels, cortical thickness and cortical bone strength with no effect on trabecular bone volume fraction. Mechanistic studies demonstrated that the reduced cortical bone thickness was caused by a combination of increased bone resorption and reduced periosteal bone formation. In conclusion, WNT16 is a crucial regulator of cortical bone thickness in young adult and old mice. We propose that new treatment strategies targeting the adult regulation of WNT16 might be useful to reduce fracture risk at cortical bone sites.

## Introduction

Osteoporosis affects hundreds of millions of people worldwide and fragility fractures cause enormous problems particularly for postmenopausal women and older men (Baron & Hesse 2012). Cortical bone is a key determinant of bone strength and non-vertebral fracture risk (Zebaze *et al*. 2010, Ohlsson *et al*. 2017). Currently available osteoporosis treatments mainly affect the trabecular bone reducing the risk of vertebral fractures by up to 70% while non-vertebral fracture risk has been improved only marginally by currently available treatments, defining an unmet medical need (Cummings *et al*. 2009, Baron & Hesse 2012). It is, therefore, of high clinical importance to increase the knowledge of the regulation of cortical bone mass.

Several large scale human genetic studies have demonstrated that the *WNT16* locus is reproducibly associated with cortical bone thickness, bone mineral density and non-vertebral fractures (Estrada *et al*. 2012, Medina-Gomez *et al*. 2012, Zheng *et al*. 2012, Garcia-Ibarbia *et al*. 2013, Koller *et al*. 2013, Hendrickx *et al*. 2014). Subsequent translational studies using global as well as cell-specific *Wnt16* inactivation in mice demonstrated that osteoblast-derived WNT16 regulates cortical bone thickness and non-vertebral fracture susceptibility (Moverare-Skrtic *et al*. 2014). Osteoblast-derived WNT16 affects cortical bone by inhibiting cortical osteoclast formation both by inhibiting RANK-signaling in osteoclast progenitor cells and by enhancing *Opg* expression in osteoblasts (Moverare-Skrtic *et al*. 2014). In addition, it has been described that WNT16 may also regulate periosteal bone formation rate (Wergedal *et al*. 2015).

Although very informative, the previous experimental studies using mouse models with life-long global or cell-specific *Wnt16* inactivation could not exclude the possibility that the effect of *Wnt16* inactivation on cortical bone thickness might be caused by early developmental effects (Moverare-Skrtic *et al*. 2014, Wergedal *et al*. 2015). These studies, therefore, could not separate between developmental effects of WNT16 and its effects on adult bone metabolism. As we want to determine the possible usefulness of WNT16 as an osteoporosis drug target, it is crucial to determine if WNT16 exerts important effects on cortical bone homeostasis in adult and old mice. Thus, if WNT16 only would have an effect during early development, but not in adult or old mice, this would mean that WNT16 never will become be an interesting osteoporosis drug target as osteoporosis treatment is given to relatively old subjects. Therefore, to evaluate the effect of WNT16 specifically on adult cortical bone homeostasis, we developed a mouse model with tamoxifen-inducible efficient global *Wnt16* inactivation (Hayashi & McMahon 2002) and determined the effects of WNT16 on cortical bone mass in young adult and old mice. This mouse model is more similar to a systemic modulation of WNT16 activity than the previous cell-specific mouse models that we have used for detailed mechanistic studies of the effect of WNT16 on cortical bone. In these previous studies, we used *Runx2-Cre* and *Dmp1-Cre* mouse models to demonstrate that osteoblast-derived WNT16 contributes to the regulation of cortical bone thickness (Moverare-Skrtic *et al*. 2014). To evaluate WNT16 as a possible osteoporosis drug target, one has to consider not only osteoblast-derived WNT16 but also global WNT16 expression that may exert off target side effects, arguing for the use of an inducible global *Wnt16* inactivation mouse model in the present study.

## Materials and methods

### Animals

Generation of *Wnt16* conditional knockout mice (*Wnt16*^flox/flox^) on C57BL/6N background has been described recently (Moverare-Skrtic *et al*. 2014). Briefly, exon 3 of the *Wnt16* gene is flanked by *loxP* sites in these *Wnt16*^flox/flox^ mice. The following primer pairs were used for genotyping of the presence or absence of the *loxP* sequence: 5′-CATAAAGCCAGCTGCACTGC-3′ and 5′-AAATGTGTAACCTTCACGAG-3′. To be able to delete the floxed sequence of the *Wnt16* gene in an inducible manner, we used B6.Cg-Tg(CAG-cre/Esr1*)5Amc/J (CAGGCre-ER) transgenic mice expressing a tamoxifen-inducible Cre-mediated recombination system (#004682, Jackson Laboratories (Hayashi & McMahon 2002)). To generate tamoxifen-inducible knockout mice, *Wnt16*^flox/flox^ female mice were mated with CAGGCre-ER-*Wnt16*^+/flox^ male mice. The tamoxifen-inducible offsprings, CAGGCre-ER-*Wnt16*^flox/flox^ mice, were called *Cre-Wnt16*^flox/flox^. The control mice were littermate *Wnt16*^flox/flox^ mice without CAGGCre-ER expression. The mice were housed in a standard animal facility under controlled temperature (22°C) and photoperiod (12 h of light and 12 h of darkness) and had free access to water and food pellets (RM1A, SDS Diet, UK). All experimental procedures involving animals were approved by the Ethics Committee at the University of Gothenburg and carried out in accordance with relevant guidelines.

### Tamoxifen treatment

Tamoxifen (T5648, Sigma-Aldrich) was dissolved in ethanol at a concentration of 100 mg/mL and further diluted in corn oil (C8267, Sigma-Aldrich) to a concentration of 10 mg/mL. The tamoxifen suspension was administered to the *Cre-Wnt16*^flox/flox^ mice and their *Wnt16*^flox/flox^ littermate control mice by intraperitoneal injections during four consecutive days. Ten-week-old male mice were given 0.25 mg/mouse/day or 1 mg/mouse/day, whereas 47-week-old female mice were given 1 mg/mouse/day. Four weeks after the first tamoxifen injection, blood was collected from the axillary vein under anesthesia with Ketanest/Dexdomitor vet (Pfizer/Orion Pharma AB, Animal Health, Sollentuna, Sweden) and the mice were subsequently killed by cervical dislocation. The long bones, vertebrae and soft tissues were dissected and stored for further analyses. In addition, to determine the effect of inducible *Wnt16* inactivation on cortical bone thickness in young adult female mice, 7-week-old female *Cre-Wnt16*^flox/flox^ and littermate *Wnt16*^flox/flox^ mice were given tamoxifen (1 mg/mouse/day) during four consecutive days and killed at 16 weeks of age.

### Assessment of bone parameters

#### X-ray analyses

Computed tomography (CT) scans of the cortical mid-diaphyseal and trabecular epiphyseal tibia and femur were performed using pQCT XCT Research M (version 4.5B; Norland) as described previously (Windahl *et al*. 1999, Vidal *et al*. 2000). High-resolution µCT (μCT) analyses were further performed on the femur and lumbar vertebra L5 (Moverare-Skrtic *et al*. 2014) following the guidelines of the American Society for Bone and Mineral Research (Bouxsein *et al*. 2010). Femur and vertebra were imaged with an X-ray tube voltage of 50 kV, a current of 201 μA and with a 0.5-mm aluminum filter. The scanning angular rotation was 180°, and the angular increment was 0.70°. The voxel size was 4.49 µm isotropically. NRecon (version 1.6.9) was used to perform the reconstruction after the scans. In the present study, we compared the effect of inducible *Wnt16* inactivation on cortical vs trabecular bone. To this end, we selected bone locations with essentially only cortical bone (mid-diaphyseal region of femur) or only trabecular bone (the inner part of the vertebral body as defined below) for µCT analyses. Cortical measurements using µCT were performed in the mid-diaphyseal region of femur starting at a distance of 5.2 mm from the distal growth plate and extending a further longitudinal distance of 134 µm in the proximal direction. In mice, this region of the femur has essentially no trabecular bone. The average cortical bone thickness is given. For trabecular bone measurements, not including cortical bone in the vertebra, the trabecular bone in the vertebral body caudal of the pedicles was selected for analysis within a conforming volume of interest (cortical bone excluded) commencing at a distance of 4.5 µm caudal of the lower end of the pedicles, and extending a further longitudinal distance of 328 µm in the caudal direction.

#### Static and dynamic bone histomorphometry

Femurs of the 51-week-old female mice were analyzed by PharmaTest Services, Ltd. as described previously (Moverare-Skrtic *et al*. 2014). Briefly, the mice were injected (i.p.) on day 4 (alizarin) and day 18 (calcein) before sacrifice. The femurs were fixed in 4% paraformaldehyde, dehydrated in 70% EtOH and embedded in methyl methacrylate. The femurs were sectioned in the transverse plane and unstained 200 μm-thick sections were analyzed for static and dynamic parameters. All parameters were measured using the OsteoMeasure histomorphometry system (OsteoMetrics, Decatur, GA, USA) following the guidelines of the American Society for Bone and Mineral Research (Dempster *et al*. 2013).

### Biomechanical strength analyses

Humerus was loaded by three-point bending test using a mechanical testing machine (Instron 3366, Instron, Norwood, MA, USA) (Wu *et al*. 2016). The loading speed was 0.155 mm/s and the span length was 4.5 mm. Based on the computer recorded load deformation raw data curves, produced by Bluehill 2 software v2.6 (Instron), the results were calculated with custom-made Excel macros.

### Real-time PCR

RNA was isolated from gonadal fat, liver and cortical bone (femur) using TRIzol reagent (Sigma) followed by the RNeasy Mini Kit (Qiagen). Amplifications were performed using the StepOnePlus Real-Time PCR System (PE Applied Biosystems) using Assay-on-Demand primer and probe sets (PE Applied Biosystems), labeled with the reporter fluorescent dye FAM. Predesigned primers and a probe labeled with the reporter fluorescent dye VIC, specific for 18S ribosomal RNA, were included in the reaction as an internal standard. The assay identification numbers were* Wnt16* Mm00446420_m1, Catepsin K (*CatK*) Mm00484036_m1, *Opg* (osteoprotegerin, *Tnfrsf11b*) Mm00435452_m1, and *Rankl (Tnfsf11)* Mm00441908_m1.

### Power calculation, blinding of investigators and randomization of mouse samples

The predesigned primary endpoint in the mouse studies was to record the effect of inducible *Wnt16* inactivation on cortical bone thickness. Our power analysis suggested that when using eight WT and eight *Wnt16*-inactivated mice, we would have 80% power to detect a biological significant effect with a 1.51 s.d. change in cortical bone thickness at a two-sided alpha level of 0.05. Therefore, we aimed to use at least eight mice per group in the different mouse studies. All *in vivo* experiments and subsequent assessments of the outcomes from these experiments were done in totally blinding of the investigators. No experiments requiring randomization of sample groups were performed.

### Statistical analyses

Values are given as mean ± s.e.m. The statistical difference between *Cre-Wnt16*^flox/flox^ and *Wnt16*^flox/flox^ mice was calculated using Student’s *t* test. If data were not normally distributed, they were log-transformed before statistical analyses. Pearson’s correlation coefficient (*r*) was calculated between cortical thickness and *Wnt16* mRNA expression. A *P* value of <0.05 was considered statistically significant.

## Results

### Inducible inactivation of the *Wnt16* gene

**Figure 1:**
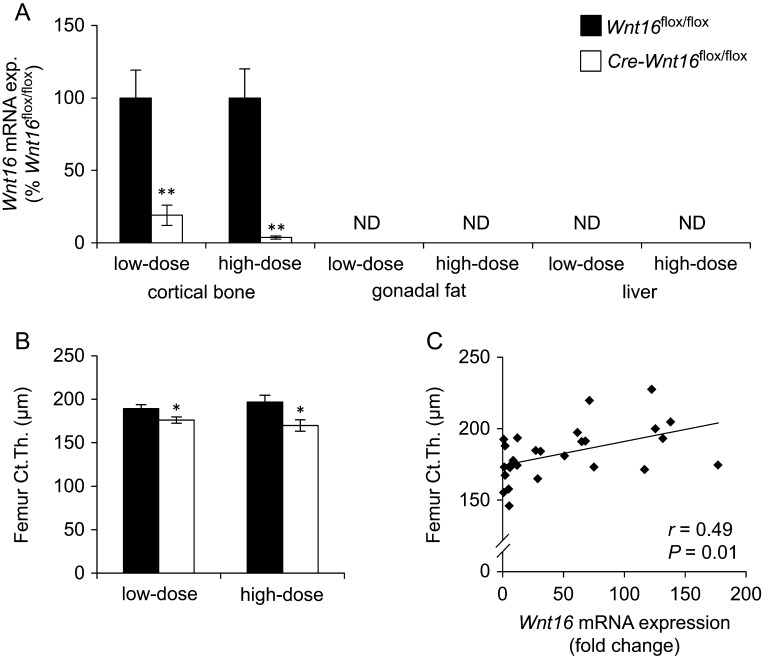
Inducible inactivation of *Wnt16* in young adult male mice reduces cortical bone thickness. Fourteen-week-old male *Cre-Wnt16*^flox/flox^ (*n* = 7) and littermate *Wnt16*^flox/flox^ (*n* = 6) control mice treated for four consecutive days with either low-dose (0.25 mg/mouse/day) or high-dose (1 mg/mouse/day) tamoxifen at the age of ten weeks. (A) Expression level of *Wnt16* mRNA in cortical bone of femur, gonadal fat and liver. (B) Cortical bone thickness of femur as analyzed using μCT. ND, not detectable. Values are given as mean ± s.e.m. (*Wnt16*^flox/flox^ low-dose, *n* = 6, high-dose *n* = 7; *Cre-Wnt16*^flox/flox^ low and high dose, *n* = 7). *P < 0.05, **P < 0.01, Student’s *t* test, *Cre-Wnt16*^flox/flox^ vs *Wnt16*^flox/flox^ control mice. (C) Correlation between *Wnt16* mRNA levels in cortical bone of femur and cortical bone thickness in the femur diaphysis. Pearson’s correlation coefficient is given.

Our expression analyses confirmed that *Wnt16* is abundantly expressed in cortical bone, whereas no detectable expression was observed in gonadal fat or liver (Fig. 1A). To assess the importance of WNT16 for the regulation of cortical bone thickness in adult mice, we developed a mouse model with inducible inactivation of *Wnt16*. To this end, we bred our recently developed mouse model having exon 3 of *Wnt16* flanked by *loxP* sites (*Wnt16*^flox/flox^ (Moverare-Skrtic *et al*. 2014)) with CAGGCre-ER transgenic mice (Hayashi & McMahon 2002) expressing a tamoxifen-inducible Cre-mediated recombination system. When comparing *Cre-Wnt16*^flox/flox^ mice with *Wnt16*^flox/flox^ littermate mice, we observed that the presence of the tamoxifen-inducible Cre-mediated recombination system without tamoxifen treatment did not influence the skeleton (Supplementary Table 1, see section on supplementary data given at the end of this article). Next, we evaluated the ability of tamoxifen to delete the floxed exon 3 of the *Wnt16* gene in young adult mice with the tamoxifen-inducible Cre recombinase. Two different doses of tamoxifen were administered daily i.p. for four consecutive days to 10-week-old *Cre-Wnt16*^flox/flox^ and *Wnt16*^flox/flox^ mice, and the mice were examined four weeks later. The low-dose (0.25 mg/mouse/day) and high-dose (1 mg/mouse/day) tamoxifen treatment reduced the *Wnt16* mRNA levels in cortical bone by 80.9 ± 7.0% (*P* < 0.01) and 96.3 ± 1.1% (*P* < 0.01), respectively in *Cre-Wnt16*^flox/flox^ mice compared with *Wnt16*^flox/flox^ mice (Fig. 1A). The inducible inactivation of *Wnt16* did not affect body weight, weights of liver or gonadal fat, or the lengths of femur or tibia (Table 1).

**Table 1:** Body characteristics of young adult male *Cre-Wnt16*^flox/flox^ and* Wnt16*^flox/flox^ mice.

	Low-dose tamoxifen		High-dose tamoxifen	
	*Wnt16*^flox/flox^ (*n* = 6)	*Cre-Wnt16*^flox/flox^ (*n* = 7)	*Wnt16*^flox/flox^ (*n* = 7)	*Cre-Wnt16*^flox/flox^ (*n* = 7)
Body weight (g)	31.1 ± 0.5	30.1 ± 1.4	29.2 ± 1.3	32.0 ± 1.8
Liver weight/body weight (%)	4.30 ± 0.08	4.37 ± 0.09	4.45 ± 0.14	4.87 ± 0.16
Gonadal fat weight/body weight (%)	1.95 ± 0.24	2.20 ± 0.35	1.87 ± 0.33	2.56 ± 0.38
Femur length (mm)	16.4 ± 0.11	16.0 ± 0.20	16.3 ± 0.17	16.2 ± 0.18
Tibia length (mm)	19.0 ± 0.06	18.7 ± 0.11	19.0 ± 0.08	18.7 ± 0.14
Tibia cortical thickness (μm)	232 ± 7	216 ± 4*	228 ± 6	199 ± 7**

Body characteristics of 14-week-old *Cre-Wnt16*^flox/flox^ and* Wnt16*^flox/flox^ male mice treated with low-dose (0.25 mg/mouse/day) or high-dose (1 mg/mouse/day) tamoxifen during four consecutive days at the age of 10 weeks. Values are given as mean ± s.e.m.

*P < 0.056, **P < 0.01, Student’s *t* test, *Cre-Wnt16*^flox/flox^ vs *Wnt16*^flox/flox^ control mice.

### Inducible inactivation of *Wnt16* in young adult mice reduces cortical bone thickness

We next evaluated if the inducible* Wnt16* inactivation affected cortical bone thickness in the long bones of young adult mice. Both inducible *Wnt16* inactivation using low-dose (−7.0 ± 1.9%; *P* < 0.05) and high-dose (−13.7 ± 3.3%; *P* < 0.05) tamoxifen reduced the cortical bone thickness in the mid-diaphyseal region of the femur in male *Cre-Wnt16*^flox/flox^ mice compared with male *Wnt16*^flox/flox^ mice (Fig. 1B). Cortical cross-sectional bone area was also reduced by inducible *Wnt16* inactivation (Supplementary Table 2). A similar reduction of the cortical bone thickness by inducible *Wnt16* inactivation was observed in the tibia (low-dose tamoxifen, −7.0 ± 1.7%, *P* = 0.056; high-dose tamoxifen, −12.6 ± 3.0%, *P* < 0.01) of male *Cre-Wnt16*^flox/flox^ mice compared with male *Wnt16*^flox/flox^ mice (Table 1). Interestingly, femur cortical bone thickness was directly associated with the *Wnt16* mRNA expression levels in cortical bone of femur (Pearson’s *r* = 0.49, *P* = 0.01) when evaluated in all mice, supporting a role of WNT16 in the regulation of cortical bone thickness (Fig. 1C). The finding that inducible *Wnt16* inactivation reduced cortical bone thickness in young adult male mice was replicated in young adult female mice. Inducible *Wnt16* inactivation reduced femur cortical thickness (−18.7 ± 6.4%; *P* < 0.01) in 16-week-old female *Cre-Wnt16*^flox/flox^ mice compared with female *Wnt16*^flox/flox^ mice.

Analyses of the trabecular bone in the lumbar vertebrae L5 demonstrated that the reduced cortical bone thickness in young adult mice with inducible inactivation of *Wnt16* was not associated with reduced trabecular bone volume fraction (BV/TV), trabecular number (Tb.N), trabecular thickness (Tb.Th) or trabecular separation (Tb.Sp) (Table 2). In contrast, the high-dose but not the low-dose tamoxifen treatment modestly increased trabecular BV/TV in male *Cre-Wnt16*^flox/flox^ mice compared with male *Wnt16*^flox/flox^ mice (Table 2).

**Table 2:** Trabecular bone characteristics of lumbar vertebra L5 of tamoxifen-treated *Cre-Wnt16*^flox/flox^ and* Wnt16*^flox/flox^ mice.

	*Wnt16*^flox/flox^	*Cre-Wnt16*^flox/flox^
14-week-old male mice (low-dose tamoxifen)	*n* = 6	*n* = 7
Bone volume/total volume (BV/TV; %)	29.5 ± 1.6	27.8 ± 0.6
Trabecular thickness (Tb.Th; µm)	48.7 ± 1.1	46.7 ± 0.9
Trabecular number (Tb.N; /mm)	6.1 ± 0.3	6.0 ± 0.1
Trabecular separation (Tb.Sp; µm)	123 ± 5.7	125 ± 2.9
14-week-old male mice (high-dose tamoxifen)	*n* = 6	*n* = 7
Bone volume/total volume (BV/TV; %)	25.7 ± 1.1	30.0 ± 1.3*
Trabecular thickness (Tb.Th; µm)	46.8 ± 1.9	49.6 ± 1.1
Trabecular number (Tb.N; /mm)	5.3 ± 0.3	6.0 ± 0.2
Trabecular separation (Tb.Sp; µm)	141 ± 5.0	126 ± 4.6
51-week-old female mice (high-dose tamoxifen)	*n* = 11	*n* = 9
Bone volume/total volume (BV/TV; %)	24.8 ± 2.9	20.6 ± 2.2
Trabecular thickness (Tb.Th; µm)	54.2 ± 2.1	55.1 ± 1.7
Trabecular number (Tb.N; /mm)	4.6 ± 0.6	3.8 ± 0.5
Trabecular separation (Tb.Sp; µm)	86.2 ± 5.1	90.9 ± 5.2

Trabecular bone µCT analyses of lumbar vertebra L5 in 14-week-old *Cre-Wnt16*^flox/flox^ and* Wnt16*^flox/flox^ male mice treated with low-dose (0.25 mg/mouse/day) or high-dose (1 mg/mouse/day) tamoxifen during four consecutive days at 10 weeks of age and 51-week-old *Cre-Wnt16*^flox/flox^ and* Wnt16*^flox/flox^ female mice treated with high-dose (1 mg/mouse/day) tamoxifen during four consecutive days at the age of 47 weeks. Values are given as mean ± s.e.m.

*P < 0.05, Student’s *t* test, *Cre-Wnt16*^flox/flox^ vs *Wnt16*^flox/flox^ control mice.

### Inducible inactivation of *Wnt16* in older female mice reduces cortical bone thickness

**Figure 2:**
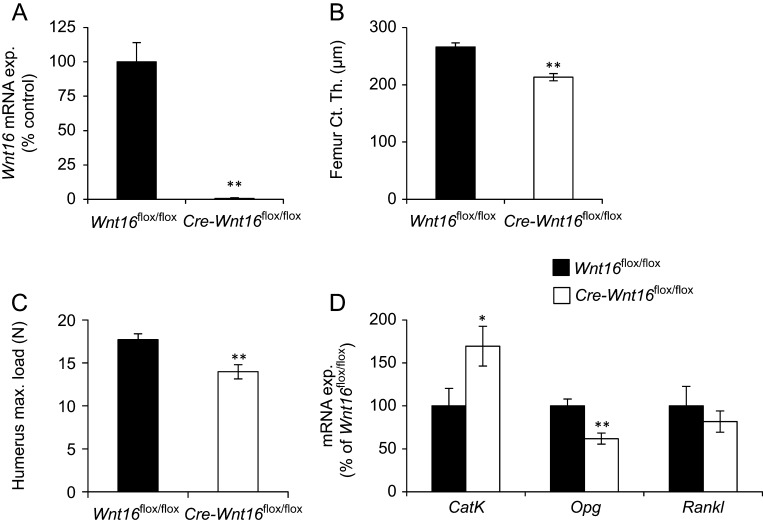
Inducible inactivation of *Wnt16* in older female mice reduces cortical bone thickness. 51-week-old female *Cre-Wnt16*^flox/flox^ and littermate *Wnt16*^flox/flox^ control mice treated with tamoxifen (1 mg/mouse/day) for four consecutive days at 47 weeks of age. (A) *Wnt16* mRNA levels in cortical diaphyseal bone of femur. (B) Cortical thickness of the mid-diaphysis of femur as analyzed using µCT and (C) maximal (max.) load at failure determined by three-point bending of humerus. (D) mRNA levels of *CatK*, *Opg* and *Rankl* in cortical diaphyseal bone of femur. Values are given as mean ± s.e.m. (*Wnt16*^flox/flox^ *n* = 11; *Cre-Wnt16*^flox/flox^ *n* = 9). **P < 0.01, Student’s *t* test, *Cre-Wnt16*^flox/flox^ vs *Wnt16*^flox/flox^ control mice.

We next evaluated if WNT16 regulates cortical bone thickness also in older mice. For inducible *Wnt16* inactivation, both *Cre-Wnt16*^flox/flox^ and *Wnt16*^flox/flox^ mice were treated by tamoxifen (1 mg/mouse/day, i.p. for four consecutive days) four weeks before killing at 51 weeks of age. Tamoxifen treatment almost completely inactivated *Wnt16* expression in cortical bone (−99.3 ± 0.4%; *P* < 0.01) in the *Cre-Wnt16*^flox/flox^ mice compared with* Wnt16*^flox/flox^ mice (Fig. 2A). The inducible *Wnt16* inactivation did not affect body weight, weights of liver or gonadal fat or the lengths of femur or tibia (Table 3). In contrast, the inducible *Wnt16* inactivation reduced the cortical thickness of femur (−19.8 ± 2.4%, *P* < 0.01; Fig. 2B) and tibia as analyzed by CT (−14.5 ± 3.2%, *P* < 0.01; Table 3) in the *Cre-Wnt16*^flox/flox^ mice compared with *Wnt16*^flox/flox^ mice. Cortical cross-sectional bone area was also reduced in the femur by inducible *Wnt16* inactivation (Supplementary Table 3). Static histomorphometric analyses of femur confirmed a reduced cortical bone area (−17.6 ± 6.6%, *P* < 0.05) and cortical thickness (−17.0 ± 5.5%, *P* < 0.05) in the *Cre-Wnt16*^flox/flox^ mice compared with *Wnt16*^flox/flox^ mice (Table 4). In contrast, no significant change was observed for total bone area or marrow cavity area (Table 4 and Supplementary Table 3). Three-point bending analysis of the humerus diaphysis revealed a substantial reduction (−21.1 ± 4.6%; *P* < 0.05) in maximal load at failure in the tamoxifen-treated *Cre-Wnt16*^flox/flox^ mice as compared to the tamoxifen-treated *Wnt16*^flox/flox^ mice, demonstrating that the decreased cortical bone thickness reduced the bone strength (Fig. 2C). The inducible *Wnt16* inactivation did not affect trabecular bone parameters in the older female mice (Table 2).

**Table 3:** Body characteristics of old female tamoxifen-treated *Cre-Wnt16*^flox/flox^ and* Wnt16*^flox/flox^ mice.

	*Wnt16*^flox/flox^ (*n* = 11)	*Cre-Wnt16*^flox/flox^ (*n* = 9)
Body weight (g)	33.7 ± 1.4	31.3 ± 2.9
Liver weight/body weight (%)	3.54 ± 0.36	3.74 ± 0.64
Gonadal fat weight/body weight (%)	5.43 ± 0.58	4.64 ± 0.87
Femur		
Bone length (mm)	16.6 ± 0.11	16.8 ± 0.11
Periosteal circumference (mm)	4.82 ± 0.08	4.62 ± 0.11
Endosteal circumference (mm)	3.15 ± 0.09	3.28 ± 0.10
Tibia		
Bone length (mm)	18.8 ± 0.11	18.8 ± 0.12
Periosteal circumference (mm)	4.20 ± 0.05	4.09 ± 0.10
Endosteal circumference (mm)	2.59 ± 0.06	2.71 ± 0.10
Cortical thickness (µm)	256 ± 9	219 ± 8**

Body characteristics of 51-week-old female *Cre-Wnt16*^flox/flox^ and* Wnt16*^flox/flox^ mice treated with tamoxifen (1 mg/mouse/day) during four consecutive days at the age of 47 weeks. Values are given as mean ± s.e.m.

**P < 0.01, Student’s *t* test, *Cre-Wnt16*^flox/flox^ vs *Wnt16*^flox/flox^ control mice.

**Table 4:** Histomorphometric analyses of cortical bone of old female tamoxifen-treated *Cre-Wnt16*^flox/flox^ and* Wnt16*^flox/flox^ mice.

	*Wnt16*^flox/flox^ (*n* = 11)	*Cre-Wnt16*^flox/flox^ (*n* = 8)
Static histomorphometry		
Total bone area (B.Ar; mm^2^)	1.90 ± 0.09	1.77 ± 0.10
Marrow cavity area (Ma.Ar; mm^2^)	0.79 ± 0.05	0.85 ± 0.06
Cortical bone area (Ct.Ar; mm^2^)	1.12 ± 0.05	0.92 ± 0.07*
Cortical width (Ct.Wi; mm)	0.28 ± 0.01	0.23 ± 0.01*
Dynamic histomorphometry		
Periosteal histomorphometry		
Mineral surface/bone surface (MS/BS; %)	47.5 ± 6.3	28.7 ± 3.0*
Mineral apposition rate (MAR; µm/day)	0.82 ± 0.08	0.64 ± 0.10
Bone formation rate (BFR; mm^3^/mm^2^/year)	153 ± 32	71.6 ± 18*
Endocortical histomorphometry		
Mineral surface/bone surface (MS/BS; %)	83.7 ± 5.9	64.7 ± 7.4
Mineral apposition rate (MAR; µm/day)	1.20 ± 0.09	1.33 ± 0.14
Bone formation rate (BFR; mm^3^/mm^2^/year)	374 ± 49	334 ± 61

Histomorphometric analyses of femur cortical bone of 51-week-old female *Cre-Wnt16*^flox/flox^ and* Wnt16*^flox/flox^ mice treated with tamoxifen at the age of 47 weeks. Values are given as mean ± s.e.m.

*P < 0.05, Student’s *t* test, *Cre-Wnt16*^flox/flox^ vs *Wnt16*^flox/flox^ control mice.

### *Wnt16* regulates both periosteal bone formation and bone resorption in older female mice

To investigate the mechanisms by which inducible *Wnt16* inactivation reduces cortical bone thickness in older female mice, dynamic histomorphometric analyses of femur were performed (Table 4). Inducible *Wnt16* inactivation decreased periosteal bone formation rate (BFR; −53.1 ± 11.8%, *P* < 0.05), mainly due to reduced mineralized bone surface (MS/BS; −39.6 ± 6.3%, *P* < 0.05) (Table 4). In contrast, at the endocortical surface, bone formation was not affected by inducible *Wnt16* inactivation (Table 4). Similar to what previously have been described for global life-long *Wnt16* inactivation and osteoblast-lineage-specific *Wnt16* inactivation (Moverare-Skrtic *et al*. 2014), the mRNA levels of the anti-osteoclastogenic factor *Opg* in cortical bone were reduced by inducible *Wnt16* inactivation (Fig. 2D). In addition, inducible *Wnt16* inactivation significantly increased mRNA levels of the osteoclast marker *CatK* in cortical bone (+69.5 ± 23.1%, *P* < 0.05; Fig. 2D).

## Discussion

Cortical bone mass is a major determinant of bone strength and non-vertebral fracture risk (Zebaze *et al*. 2010, Ohlsson *et al*. 2017). Although recent human genetic association studies and experimental studies using mouse models with life-long inactivation of *Wnt16* have established that WNT16 is a crucial regulator of cortical bone thickness and non-vertebral fracture risk (Estrada *et al*. 2012, Medina-Gomez *et al*. 2012, Zheng *et al*. 2012, Garcia-Ibarbia *et al*. 2013, Koller *et al*. 2013, Hendrickx *et al*. 2014, Moverare-Skrtic *et al*. 2014), it has not been possible to determine if WNT16 exerts its effect on cortical bone mainly during development and growth or if WNT16 also is crucial for adult cortical bone homeostasis. We, herein, developed an inducible *Wnt16* inactivated mouse model and demonstrated that WNT16 exerts important effects on cortical bone homeostasis both in young adult and old mice.

In the present study, we developed an inducible mouse model where the *Wnt16* gene is normally expressed until it is inactivated by tamoxifen treatment (Hayashi & McMahon 2002). To this end, we used an inducible Cre-loxP transgenic system where the Cre-ER fusion protein is sequestered in the cytoplasm. In the presence of tamoxifen, Cre-ER translocates to the nucleus and drives recombination of the floxed target gene (Feil *et al*. 1997). Although this Cre transgenic system is well studied and known to have low background Cre activity in the absence of an inducer, previous reports indicate that the use of the tamoxifen-inducible Cre-loxP system is not without potential drawbacks (Manolagas & Kronenberg 2014, Jardí *et al*. 2017, Patel *et al*. 2017). Using correct controls are therefore fundamental. In this study, we demonstrated that the Cre recombinase expressed by the transgenic mice is not able to inactivate the floxed *Wnt16* gene before tamoxifen is administered, validating the model to be inducible. The efficiency of the recombination are affected by many parameters such as the particular genomic location, the distance between the loxP sites and the ability of tamoxifen to reach different target organs (Feil *et al*. 2009). Using cell-specific *Wnt16* inactivation, we have previously demonstrated that WNT16 in cortical bone is osteoblast derived and that the effect of WNT16 on cortical bone thickness is completely mediated by osteoblast-derived WNT16 (Moverare-Skrtic *et al*. 2014). Therefore, to determine the role of WNT16 for adult cortical bone metabolism, it is important to achieve an efficient inducible inactivation of *Wnt16* in cortical bone, and in the present study, the efficiency of recombination of the *Wnt16* gene in the cortical bone was almost complete when induced by tamoxifen treatment both in young adult and in old mice.

Tamoxifen, used for the inducible inactivation of *Wnt16* in the present study, is a selective estrogen receptor modulator (SERM) that has been reported to affect the skeleton (Perry *et al*. 2005, Zhong *et al*. 2015). The possible confounding effects of tamoxifen on the skeleton were avoided by the fact that the control (*Wnt16*^flox/flox^) mice received the same dose of tamoxifen as the inducible *Wnt16*-knockout mice (*Cre-Wnt16*^flox/flox^) and, in addition, there was a 3.5-week wash-out period between the last tamoxifen treatment and the analyses of the skeletal phenotype. However, we cannot completely exclude the possibility that the tamoxifen treatment might have blunted or confounded some of the effects of WNT16 on bone metabolism in the present study.

The risk of osteoporosis and osteoporosis-related fractures is increasing by age and it is, therefore, of importance that candidate osteoporosis drug targets should be functional also during aging. A drawback with life-long gene inactivation using standard knockout mouse models is that it is difficult to separate effects on development and growth from effects being crucial also during aging. As we aimed to determine the possible usefulness of WNT16 as an osteoporosis drug target, it was crucial to determine if WNT16 exerts important effects on cortical bone homeostasis in adult and old mice. Thus, if WNT16 would only have had an effect during early development but not in adult mice, this would mean that WNT16 never will be an interesting osteoporosis drug target as the osteoporosis treatment should be effective in relatively old patients with osteoporosis.

WNT16 belongs to the WNT protein family and some WNTs are established regulators of skeletal development (Baron & Kneissel 2013). WNT signaling has been reported to affect all aspects of skeletal development, including craniofacial, limb and joint formation. In addition, mutations in several members of the WNT signaling pathways result in skeletal malformations in humans and mice (Balemans *et al*. 2001, 2002, Brunkow *et al*. 2001, Staehling-Hampton *et al*. 2002, Loots *et al*. 2005, Kim *et al*. 2011, Laine *et al*. 2013) and based on these findings, we could not exclude that the effect of WNT16 on the skeleton in previous studies also may be dependent on early developmental effects. As the *Wnt16* inactivation used in the present study was inducible, it was possible to determine the role of WNT16 specifically for adult cortical bone homeostasis both in young adult (14-week-old) and in old (51-week-old) mice. We clearly demonstrated that WNT16 is crucial for cortical bone homeostasis both in young adult and old mice, supporting the notion that treatment strategies targeting the regulation of WNT16 might be useful to reduce fracture risk at cortical bone sites in old patients with osteoporosis. In addition, we demonstrated that inducible* Wnt16* inactivation reduced cortical bone thickness in both young adult male and young adult female mice, suggesting that WNT16 is a crucial regulator of adult cortical bone thickness in both male and female mice.

Previous studies using several different mouse models with life-long global or cell-specific *Wnt16* inactivation have collectively shown that osteoblast-derived WNT16 is a crucial regulator of cortical bone thickness and cortical bone strength when evaluated before sexual maturation and at young adult age (Zheng *et al*. 2012, Moverare-Skrtic *et al*. 2014, Wergedal *et al*. 2015). In the present study, we observed a very similar cortical bone phenotype of reduced cortical bone thickness and strength when *Wnt16* was inactivated as late as in nearly one-year-old mice. Thus, WNT16 is a crucial regulator of cortical bone homeostasis during the entire lifespan in mice. In the present study, we also observed that cortical bone thickness was directly associated with the *Wnt16* mRNA expression levels in cortical bone of femur supporting a role of WNT16 in the regulation of cortical bone thickness.

In contrast to the reduction of cortical bone thickness, the trabecular bone volume fraction was not decreased in the young adult or old mice with inducible *Wnt16* inactivation. Inducible *Wnt16* inactivation did not affect trabecular bone volume fraction in old mice while it actually modestly increased this parameter in young adult mice given high dose tamoxifen treatment. One may speculate that, in an attempt to maintain overall bone strength, the reduction in cortical bone thickness resulted in a compensatory increase in trabecular bone volume fraction in young adult mice.

The reduced cortical bone thickness in the old *Wnt16*-inactivated mice was associated with parameters reflecting increased bone resorption (increased *CatK* and reduced *Opg* mRNA levels in cortical bone) and reduced periosteal bone formation, supporting previous studies using mouse models with life-long global inactivation of *Wnt16* (Moverare-Skrtic *et al*. 2014, Wergedal *et al*. 2015). The periosteal bone formation was reduced as a result of a reduction in mineralized bone surface while mineral apposition rate was unaffected in mice with inducible *Wnt16* inactivation, suggesting a lower number of periosteal cells rather than a lower activity per cell.

In conclusion, WNT16 is a crucial regulator of cortical bone thickness in young adult and old mice. We propose that new treatment strategies targeting the adult regulation of WNT16 might be useful to reduce fracture risk at cortical bone sites.

## Supplementary Material

Supplemental Table 1. Body characteristics of untreated *Cre-Wnt16*^flox/flox^ and *Wnt16*^flox/flox^ male mice

Supplemental Table 2. Cortical bone characteristics of femur of tamoxifen-treated *Cre-Wnt16*^flox/flox^ and *Wnt16*^flox/flox^ mice

Supplemental Table 3. Cortical bone characteristics of femur of tamoxifen-treated *Cre-Wnt16*^flox/flox^ and *Wnt16*^flox/flox^ mice

## Funding

This study was supported by the Swedish Research Council, the Swedish Foundation for Strategic Research, the European Calcified Tissue Society, the ALF/LUA research grant from the Sahlgrenska University Hospital, the IngaBritt and Arne Lundberg Foundation, the Torsten and Ragnar Söderberg’s Foundation, the Knut and Alice Wallenberg Foundation and the Novo Nordisk Foundation.
