# Supplementary material for: Inducible Wnt16 inactivation: WNT16 regulates cortical bone thickness in adult mice
Source: J Endocrinol. 2018 Mar 12;237(2):113–22. doi: 10.1530/JOE-18-0020 (PMC5886037; doi:10.1530/JOE-18-0020)
Supplement: Supplemental Table 1. [file joe-237-113-t001.pdf]

Supplemental Table 1. Body characteristics of untreated *Cre-Wnt16*$^{flox/flox}$ and *Wnt16*$^{flox/flox}$ male mice

	Wnt16$^{flox/flox}$ **n = 7**	***Cre-Wnt16***$^{flox/flox}$ **n = 7**
Body weight (g)	31.0 ± 2.2	29.5 ± 1.0
Femur		
Bone length (mm)	16.1 ± 0.1	15.8 ± 0.1
Trabecular BMD (mg/cm^3)	347 ± 39	339 ± 24
Cortical thickness (µm)	208 ± 7	207 ± 4
Tibia		
Bone length (mm)	18.3 ± 0.1	18.4 ± 0.1
Trabecular BMD (mg/cm^3)	297 ± 31	296 ± 13
Cortical thickness (µm)	220 ± 7	223 ± 4

Body characteristics of 14-week-old untreated *Cre-Wnt16*$^{flox/flox}$ and *Wnt16*$^{flox/flox}$ male mice as measured by computed tomography. BMD = bone mineral density.Values are given as mean ± SEM.
