# Supplementary material for: Inducible Wnt16 inactivation: WNT16 regulates cortical bone thickness in adult mice
Source: J Endocrinol. 2018 Mar 12;237(2):113–22. doi: 10.1530/JOE-18-0020 (PMC5886037; doi:10.1530/JOE-18-0020)
Supplement: Supplemental Table 2. [file joe-237-113-t002.pdf]

**Supplemental Table 2. Cortical bone characteristics of femur of tamoxifen-treated *Cre-Wnt16*$^{flox/flox}$ and *Wnt16*$^{flox/flox}$ mice**

| | ***Wnt16*$^{flox/flox}$** | ***Cre-Wnt16*$^{flox/flox}$** |
|---|---|---|
| Low dose tamoxifen | n = 6 | n = 7 |
| Total bone area (B.Ar; mm$^2$) | 2.59 ± 0.14 | 2.21 ± 0.08* |
| Marrow cavity area (Ma.Ar; mm$^2$) | 1.50 ± 0.07 | 1.31 ± 0.06 |
| Cortical bone area (Ct.Ar; mm$^2$) | 1.09 ± 0.07 | 0.90 ± 0.02* |
| High dose tamoxifen | n = 7 | n = 7 |
| Total bone area (B.Ar; mm$^2$) | 2.26 ± 0.10 | 2.24 ± 0.10 |
| Marrow cavity area (Ma.Ar; mm$^2$) | 1.24 ± 0.08 | 1.35 ± 0.07 |
| Cortical bone area (Ct.Ar; mm$^2$) | 1.02 ± 0.04 | 0.89 ± 0.04* |

Computed tomography analyses of diaphyseal femur cortical bone in 14-week-old *Cre-Wnt16*$^{flox/flox}$ and *Wnt16*$^{flox/flox}$ male mice treated with low dose (0.25 mg/mus/day) or high dose (1 mg/mouse/day) tamoxifen during four consecutive days at 10 weeks of age. Values are given as mean ± SEM. *$P < 0.05$, Student's *t* test, *Cre-Wnt16*$^{flox/flox}$ vs. *Wnt16*$^{flox/flox}$ control mice.
