# Supplementary material for: Inducible Wnt16 inactivation: WNT16 regulates cortical bone thickness in adult mice
Source: J Endocrinol. 2018 Mar 12;237(2):113–22. doi: 10.1530/JOE-18-0020 (PMC5886037; doi:10.1530/JOE-18-0020)
Supplement: Supplemental Table 3. [file joe-237-113-t003.pdf]

Supplemental Table 3. Cortical bone characteristics of femur of tamoxifen-treated *Cre-Wnt16*$^{\mathbf{flox/flox}}$ and *Wnt16*$^{\mathbf{flox/flox}}$ mice

	Wnt16$^{\mathbf{flox/flox}}$ **n = 11**	***Cre-Wnt16***$^{\mathbf{flox/flox}}$ **n = 9**
Total bone area (B.Ar; mm^2)	2.23 ± 0.07	2.05 ± 0.09
Marrow cavity area (Ma.Ar; mm^2)	1.04 ± 0.05	1.12 ± 0.07
Cortical bone area (Ct.Ar; mm^2)	1.19 ± 0.04	0.92 ± 0.04**

Computed tomography analyses of diaphyseal femur cortical bone in 51-week-old *Cre-Wnt16*$^{\text{flox/flox}}$ and *Wnt16*$^{\text{flox/flox}}$ female mice treated with high dose (1 mg/mouse/day) tamoxifen during four consecutive days at the age of 47 weeks. Values are given as mean ± SEM. $^{**}P < 0.01$, Student's *t* test, *Cre-Wnt16*$^{\text{flox/flox}}$ vs. *Wnt16*$^{\text{flox/flox}}$ control mice.
